# Additive Manufacturing of CrFeNiTi Multi-Principal Element Alloys

**DOI:** 10.3390/ma15227892

**Published:** 2022-11-08

**Authors:** Marius Reiberg, Leonhard Hitzler, Lukas Apfelbacher, Jochen Schanz, David Kolb, Harald Riegel, Ewald Werner

**Affiliations:** 1 Institute of Materials Science, Technical University Munich, 85748 Garching, Germany; 2 Laser Application Center (LAZ), Aalen University, 73430 Aalen, Germany

**Keywords:** high entropy alloy, mechanical alloying, powder-bed fusion, materials characterization, micro-hardness

## Abstract

High entropy alloys (HEAs) and their closely related variants, called multi-principal element alloys (MPEAs), are the topic of a rather new area of research, and so far, the gathered knowledge is incomplete. This is especially true when it comes to material libraries, as the fabrication of HEA and MPEA samples with a wide variation in chemical compositions is challenging in itself. Additive manufacturing technologies are, to date, seen as possibly the best option to quickly fabricate HEA and MPEA samples, offering both the melting metallurgical and solid-state sintering approach. Within this study, CrFeNiTi MPEA samples were fabricated via laser powder-bed fusion (PBF-LB) and solid-state sintering of mechanically alloyed powder feedstock. The main emphasis is on the PBF-LB process, while solid-state sintering serves as benchmark. Within a volumetric energy density (VED) window of 50 J/mm^3^ to 83 J/mm^3^, dense samples with large defect-free sections and an average micro-hardness of 965 HV0.1 were fabricated. Clear correlations between the local chemical alloy composition and the related micro-hardness were recorded, with the main factor being the evaporation of titanium at higher VED settings through a reduction in the C14_Laves phase fraction.

## 1. Introduction

Over the last decade, high entropy alloys (HEAs) and related concepts have shown their potential for exceptional material properties in numerous research projects [1,2,3]. HEAs typically have a near equimolar ratio of at least five base elements (concentrations between 5 mol% to 35 mol%), with a single-phase microstructure stabilized by a high configurational entropy. A high lattice distortion is a characteristic of HEAs, guaranteeing a distinct solid solution hardening, which leads to a high yield strength, even at elevated temperatures [3]. The number of possible HEA compositions is indeed immense, especially when the related groups of complex-concentrated alloys (CCAs) and multi-principal element alloys (MPEAs) are also considered. AlCrFeNiTi alloys have been successfully processed via solid-state sintering and are known for their formation of a multiphase microstructure [4,5,6]. Thus, the terminology MPEA, instead of HEA, is used throughout this study.

Additive manufacturing (AM) techniques are seen as key technologies for enabling the experimental investigation of a large number of possible alloy compositions within a reasonable time frame [2]. Available technologies range from multi-step processes, utilizing binding media, followed by debinding and sintering steps, to single-step technologies, which completely melt the feedstock [7,8,9]. Thus, multi-step processes rely on solid-state sintering to achieve the microstructure of the part, analogous to traditional powder metallurgy, whereas single-step processes generate a solidification microstructure while simultaneously shaping the part.

A melting metallurgical approach becomes difficult when elements with very different melting and evaporation points are present in the alloy, thus, complicating the processability via single-step AM processes. The fabrication of metals in single-step AM processes occurs predominantly through full melting of the feedstock and as such, incorporates the melting metallurgical approach [8]. In addition, segregations can pose a challenge in cases with slow solidification rates. However, single-step processes can also be beneficial, since the dynamic flows in the melt pool can help to achieve a homogeneous mixture of alloying elements, especially in the absence of pre-alloyed powders. Within this study, the chosen approach is the mechanical alloying of metal powders to achieve the desired alloy composition, thereby combining the elements on a microscopic level by cold welding [10,11,12]. Subsequent densification of the mechanically alloyed powder is achieved by laser powder-bed fusion (PBF-LB) and solid-state sintering, with the latter serving as a reference. In PBF-LB, the melt pool is small and due to the high temperature gradients—ranging from the gas phase in the key hole, in which the laser energy is absorbed, to the solidification temperature at the melt pool boundary, to the surrounding solid material—strong flows are induced in the melt [8]. These, coupled with the fast quenching once the laser beam has moved on, restrict the segregation of the constituting elements. However, differing evaporation temperatures may lead to depletion of the elements with low evaporation temperatures. Within the single-step AM processes, PBF-LB is less sensitive to the different melting temperatures of the elements involved. In solid-state sintering, the bonding of the mechanically alloyed powder is achieved by diffusion processes, in which the differing melting temperatures affect the diffusivity, but do not cause segregations of the elements.

When developing new MPEA compositions, it is recommended to start with established high-alloy materials, as great efforts have been invested in obtaining optimized material properties. Consequently, the production of MPEA compositions with material properties achieved by high-alloyed materials is of great interest [2]. In this work, an MPEA alloy is prepared by combining elemental metal powders (chromium and titanium) with pre-alloyed powder materials L718 and W722, in an attempt to incorporate their excellent mechanical properties into the MPEA [13,14,15,16,17,18].

The MPEA was prepared by mechanical alloying with an increased nickel content and a decreased aluminum content compared to previous powder metallurgical studies [4,5,6]. Out of the five elements—Al, Cr, Fe, Ni, and Ti—aluminum has the highest risk of evaporation in the PBF-LB process. This represents a first step in changing the chemical composition of an equimolar AlCrFeNiTi composition towards that of L718. The step-by-step approach to the chemical composition of L718 was taken deliberately to investigate whether the positive material properties of MPEAs and L718 can be successfully combined. Due to their very attractive material properties, the alloys are potentially suitable for load-bearing applications in the high-temperature range, fulfilling additional requirements regarding wear and oxidation resistance [19,20,21].

## 2. Materials and Methodology

### 2.1. Base Materials and MPEA Production

Pre-alloyed, gas-atomized powders of the Ni-base alloy L718 and the Fe-base alloy W722 were combined with high-purity powders of Cr and Ti into an MPEA, named CrFeNiTi. The pre-alloyed powders L718 and W722 contained small amounts of additional elements, e.g., Al, Co, Mo, and Nb, which then became part of the overall mixture, in addition to the main elements of Cr, Fe, Ni, and Ti. Chemical compositions of the metal powders used and the mechanically-alloyed CrFeNiTi MPEA produced are listed in Table 1. The chemical composition of the CrFeNiTi MPEA was adjusted by changing the mixing ratio of the input powders used. The concentrations of Cr, Fe, and Ti were fixed at ~20 mol%, in combination with a large amount of Ni (~35 mol%). The substrate plate for the PBF-LB process was fabricated from an Ni-base alloy with high amounts of Cr and Fe (Table 1), as measured using an optical emission spectrometer (Spectromaxx-LMX06, SPECTRO Analytical Instruments GmbH, Kleve, Germany).

### 2.2. Mechanical Alloying

A Pulverisette P6 planetary ball mill (Fritsch GmbH, Idar-Oberstein, Germany) was utilized for the mechanical alloying of the CrFeNiTi MPEA. A total powder quantity of 39 g per batch was placed in a 250 mL tungsten carbide milling chamber in combination with tungsten carbide grinding balls in a ball-to-powder mass ratio of 10:1. The milling chamber was sealed in a glove box under argon atmosphere and then placed in the planetary ball mill. The grinding speed was set to 400 rpm, and the grinding time was 2 h. The grinding cycle consisted of 5 min of continuous grinding, alternated with a 20 min rest period to cool down the milling chamber. After grinding, only the loose powder in the milling chamber was used for further processing.

**Figure 1:**
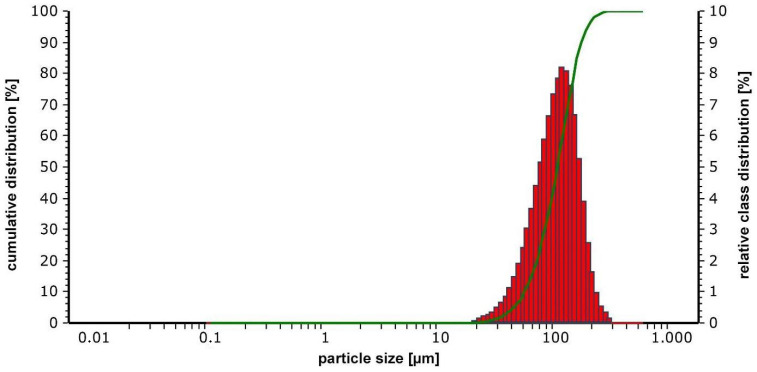
Particle size distribution of the mechanically-alloyed CrFeNiTi MPEA powder: the green curve shows the cumulative particle size distribution ranging from 0% to 100%; the red bars represent the relative percentage of the particle size classes.

The particle size distribution of the mechanically alloyed CrFeNiTi MPEA powder was determined with a Microtrac S3500 particle size analyzer (Microtrac Retsch GmbH, Haan, Germany). Demineralized water was used as dispersion medium for the laser triangulation measurement, and a 2 min ultrasonic treatment was performed to avoid powder agglomerates. The particle size distribution of the CrFeNiTi MPEA powder is displayed in Figure 1. The powder had an average powder particle size $D_{50}$ of approximately 100 µm, with $D_{10}$ and $D_{90}$ amounting to 50 µm and 160 µm, respectively.

### 2.3. Sample Fabrication

#### 2.3.1. Laser Powder-Bed Fusion

**Figure 2:**
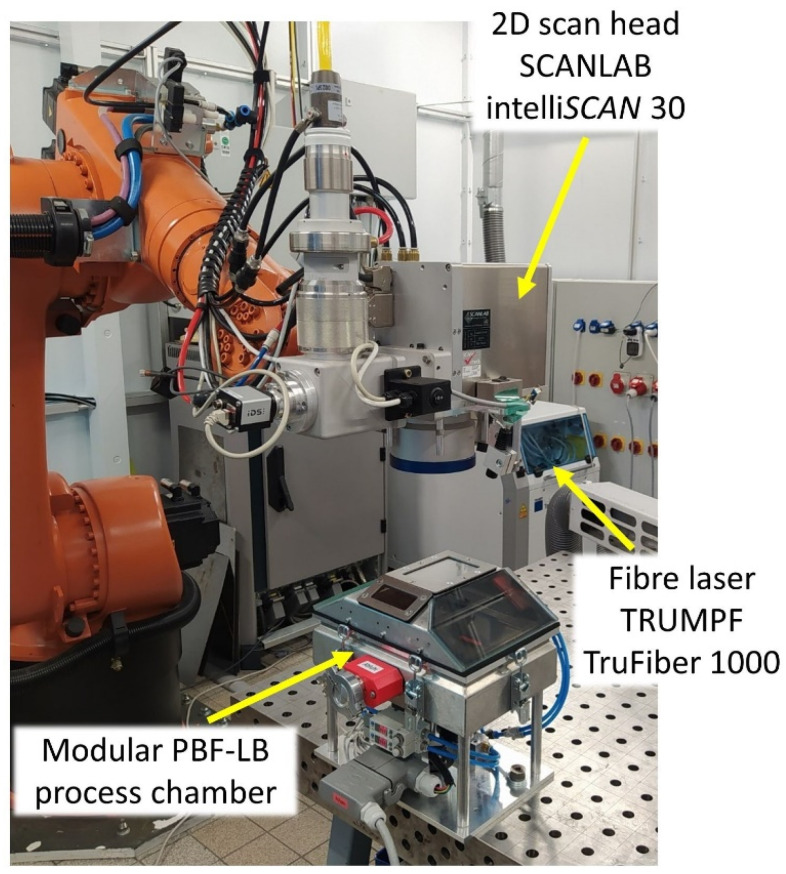
Experimental setup, including the custom-made PBF-LB chamber.

Additive manufacturing experiments were performed in a homemade modular PBF-LB process chamber (Figure 2), employing the mechanically alloyed CrFeNiTi MPEA powder. A TRUMPF TruFiber 1000 near-infrared fiber laser (TRUMPF Laser GmbH, Schramberg, Germany), with a wavelength of 1075 nm and a maximum power of 1 kW, was utilized, in combination with a SCANLAB intelli*SCAN* 30 2D scan head (SCANLAB GmbH, Puchheim, Germany). An in-depth description of the PBF-LB chamber can be found in Schanz et al. [22]. It should be emphasized that the PBF-LB chamber is designed for processing small powder quantities.

Rectangular samples with a square cross-section of 4 × 4 mm^2^ were printed on an Ni-base substrate plate (chemical composition given in Table 1), with a diameter of 40 mm and a thickness of 10 mm. The height of the fabricated samples differed within the parameter study due to the varying PBF-LB fabrication parameters. The laser power $P_{L}$ was varied between 200 W and 500 W in 100 W increments. In addition, the laser scan speed $v_{S}$ was varied between 500 mm/s and 800 mm/s in 100 mm/s increments, leading to a four-by-four array for the performed parameter study. All other parameters were kept constant throughout the PBF-LB fabrication, with a hatch distance h of 100 µm (corresponding to the laser beam diameter on the powder-bed surface) and no preheating of the substrate plate. Each layer was exposed, utilizing the bi-directional line scanning strategy with a 90° rotation increment of the scan vectors between successive layers. Argon 5.0, with a purity of 99.999%, was used as shielding gas inside the process chamber before and during sample fabrication, and the residual oxygen content was kept below 50 ppm at all times. It should be noted that the mechanically alloyed CrFeNiTi MPEA powder did not fulfil the powder quality criteria defined by Spierings and Levy [23] and Karapatis et al. [24], and the comparatively coarse feedstock necessitated a thicker than usual powder layer thickness t of 100 µm.

**Figure 3:**
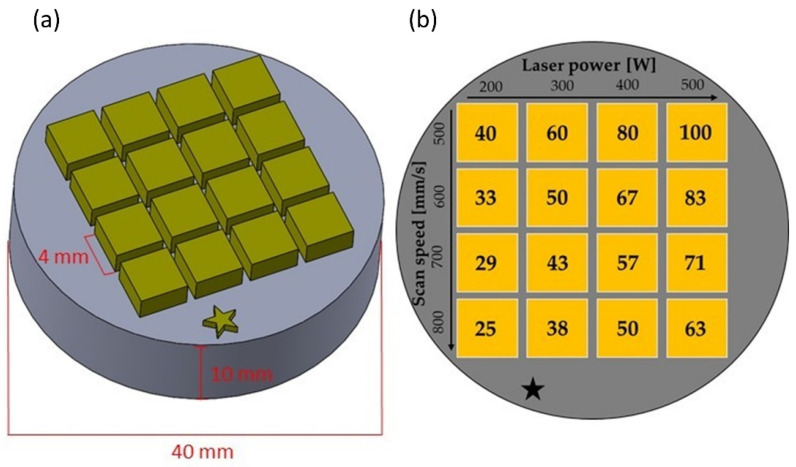
(**a**) CAD-model of the additively manufactured samples on the substrate plate. (**b**) Parameter study. The values in the yellow squares indicate the resulting volumetric energy density (VED in [J/mm^3^]) for the respective sample.

For simplicity in the nomenclature of the samples throughout this investigation, the volumetric energy density (VED) is utilized to name the PBF-LB samples (Figure 3), ranging between 25 J/mm^3^ and 100 J/mm^3^. The VED is calculated via [8]:

(1) $$VED=\frac{P_{L}}{v_{s}\;\cdot \;h\;\cdot \;t}$$

Starting with ‘MPEA_’ as a prefix, the sample produced with a laser power of 500 W and a scan speed of 500 mm/s is labelled as ‘MPEA_100′ ($VED=\frac{500\;W}{500\;\mathrm{mm}\cdot s^{-1}\;\cdot \;100\;\mu m\;\cdot \;100\;\mu m}=\frac{1\;\mathrm{Ws}}{0.1^{2}\;{\mathrm{mm}}^{3}}=100\;\frac{J}{{\mathrm{mm}}^{3}}$). The sample produced via solid-state sintering by hot isostatic pressing (HIP) is named ‘MPEA_HIP’.

#### 2.3.2. Solid-State Sintering by Hot Isostatic Pressing

**Figure 4:**
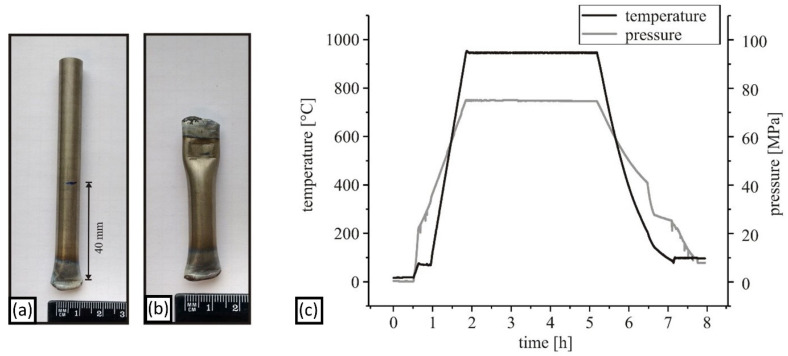
Encapsulation and solid-state sintering of CrFeNiTi MPEA powder: (**a**) V4A-tube filled with MPEA powder, (**b**) sealed capsule, (**c**) temperature and pressure cycle in HIP for densification of the encapsulated powder.

Encapsulation of the mechanically alloyed powder is necessary for its compaction by HIPing. The CrFeNiTi MPEA powder (~6 g) was filled into steel tubes (V4A, 10 mm outer diameter, 0.5 mm wall thickness, 100 mm length) under an argon atmosphere, and were then sealed (Figure 4). The capsules were subjected to a pressure of 75 MPa and a temperature of 950 °C, using a hot isostatic press (EPSI, Belgium), with high purity argon gas as the pressure medium. This environment was maintained for 4 h. The cooling rate at the end of the process was moderate and reached a maximum of 12 K/min. A detailed description of the sample preparation using HIP can be found in Reiberg et al. [6].

### 2.4. Microstructural Analyses

The samples for light microscopy (LM), scanning electron microscopy (SEM), energy dispersive X-ray spectroscopy (EDS), and hardness testing were separated from the substrate plate, by wire electrical discharge machining (WEDM). The cut samples were embedded and microsections were prepared through multiple grinding and polishing steps. Beraha III was used as the etchant. Light microscopy images were taken with a Leitz Aristomet metallographic microscope (Leica Microsystems GmbH, Wetzlar, Germany). The micro-hardness of the samples was determined using a Reichert-Jung Micro-Duromat 4000E, Leica Reichert Metaplan 2. A minimum of six measurements per sample were taken in the homogeneous section within the cross-sectional cut to determine the average Vickers hardness (HV0.1). In addition, hardness measurements were taken parallel to the build direction, in straight lines, to reveal the hardness progression within the sample and the transition to the substrate plate. The homogeneous section describes the slice in a PBF-LB sample not affected by the substrate plate—the melt pool penetration into the substrate plate results in a slight variation of the local alloy composition—and at a sufficiently large distance from the top layer. The last layers fabricated exhibit a finer microstructure due to the lack of reheating cycles (in situ heat treatment), which occur while subsequent layers are fabricated and thus, are not representative of bulk material properties [25].

A Jeol JSM-6490 field emission microscope, with a secondary electron detector and an accelerating voltage of 20 kV, was used to further analyze the microstructure. Energy dispersive spectrometry (EDS) analyses were performed with an Oxford Instruments EDS detector, INCA PentaFETx3. The EDS analyses were performed across the cross-sectional cuts, consisting of a minimum of 15 measurement points taken within the homogeneous section of each sample, and in addition, complete line scans were taken from the top layer down to the substrate plate (parallel to the build direction).

### 2.5. Thermocalc

For the calculation of the stable phases present in the CrFeNiTi MPEA, the calculation of phase diagrams (CALPHAD) method was used, as part of the Thermocalc software version 2017a, in combination with the TCHEA1 database [26].

## 3. Results and Discussion

### 3.1. PBF-LB Parameter Study

The parameter study covered a wide spectrum of potential scan speeds (500 mm/s to 800 mm/s) and laser power levels (200 W to 500 W) to shed light on the unknown process window for the CrFeNiTi MPEA. It must be mentioned that the powder quality of the mechanically alloyed powder was not ideal for PBF-LB processing; however, no pre-alloyed powder with the desired chemical composition was accessible at the time this research was conducted. For PBF processes, a powder batch of 20 µm to 63 µm in powder particle diameter is commonly employed, with the typical layer thicknesses ranging from 30 µm to 60 µm [8].

**Figure 5:**
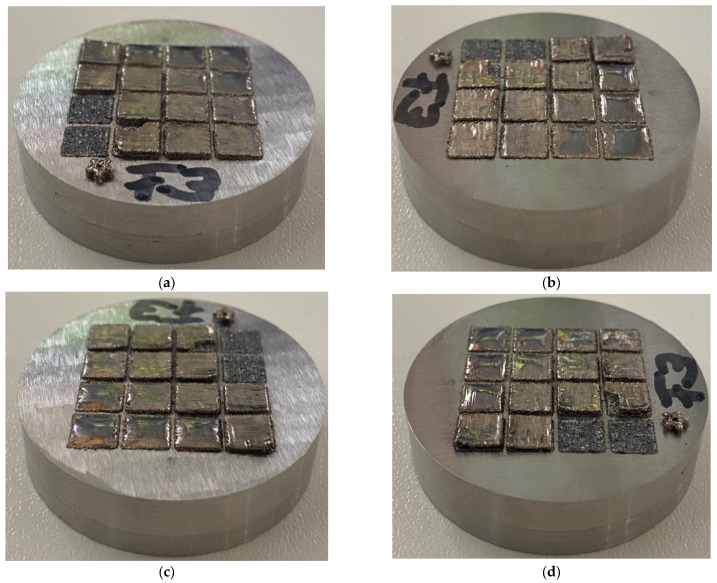
PBF-LB samples of parameter study (parameter setting detailed in Figure 3b); the star icon marks the machine front: (**a**) front view, (**b**) view from right side, (**c**) back view, (**d**) view from left side.

The majority of parameter settings were deemed unsuitable without further analyses due to visible defects (Figure 5). At a high $v_{S}$ of 700 mm/s and above, the samples exhibited large horizontal cracks and delamination, with samples fabricated with low VED completely detaching during fabrication. At lower $v_{S}$, horizontal cracking reduced, and samples fabricated with VED equal to or above 50 J/mm^3^ appeared to be of useable quality. Overall, the sample height varied greatly, and samples fabricated at higher VED were of smaller height and exhibited a more pronounced warpage.

### 3.2. Microstructural Analyses

#### 3.2.1. Sample Appearance, Defects, and Microstructure

**Figure 6:**
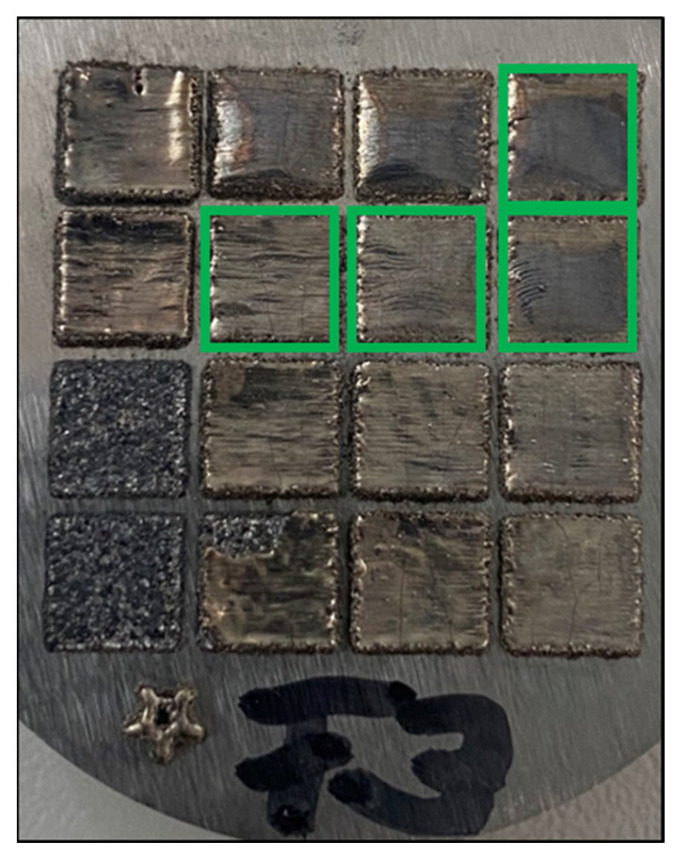
Top view of the PBF-LB samples of the parameter study (parameter settings detailed in Figure 3b). Samples marked in green were used for in-depth microstructural investigations.

**Figure 7:**
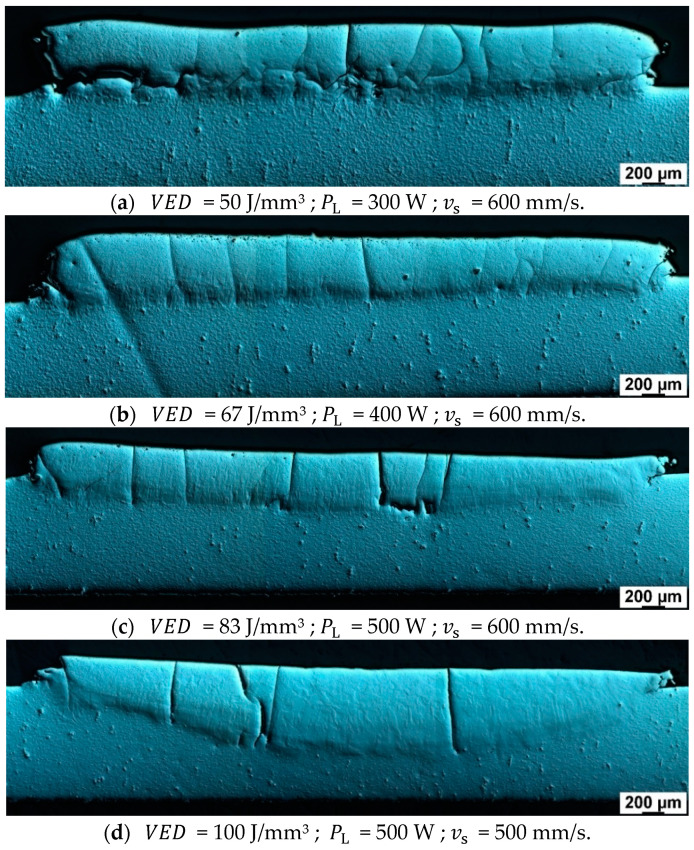
Microsections taken with the interference filter of a cross-sectional cut of: (**a**) MPEA_50, (**b**) MPEA_67, (**c**) MPEA_83, (**d**) MPEA_100.

Microsections were prepared and throughout the initial investigation, samples with noteworthy lack of fusion defects or significant keyhole porosity were ruled out. In addition to these defects, large horizontal cracks were seen as critical defects, disrupting the fabrication process due to the deterioration of the thermal conductivity towards the substrate plate, as well as the possible detachment of entire sections. Therefore, the initial set of 16 samples was reduced to four samples, which exhibited large defect-free sections, and the defects present were predominantly limited to vertical cracking (Figure 6). Vertical cracks were expected due to the absence of preheating (substrate plate preheating was not employed), coupled with the larger than usual layer thickness. Raising the build space temperature to lower cooling rates and thermal gradients is the most common approach to minimize cracking. New research also suggests that high-frequency laser power modulation is a suitable approach to prevent cracking [27]. Cross-sectional cuts of these four samples, ranging from a VED of 50 J/mm^3^ to 100 J/mm^3^, are depicted in Figure 7, and only these four samples have been considered for further microstructural investigations.

The observed remelting depth into the substrate plate directly corresponded with the VED. In case of the MPEA_50 sample, the melt pool penetrated approximately 70 µm into the substrate plate and 700 µm for the MPEA_100 sample. For the MPEA_67 sample, the remelting depth was about 140 µm and 340 µm for the MPEA_83 sample. Similarly, the achieved built-up height differed throughout the samples and ranged from approximately 300 µm (MPEA_83 sample) up to 670 µm (MPEA_50 sample). In addition, the distance between the vertical cracks also depended on the VED, with the MPEA_67 sample showing the least amount of cracking, as well as the most even top surface.

**Figure 8:**
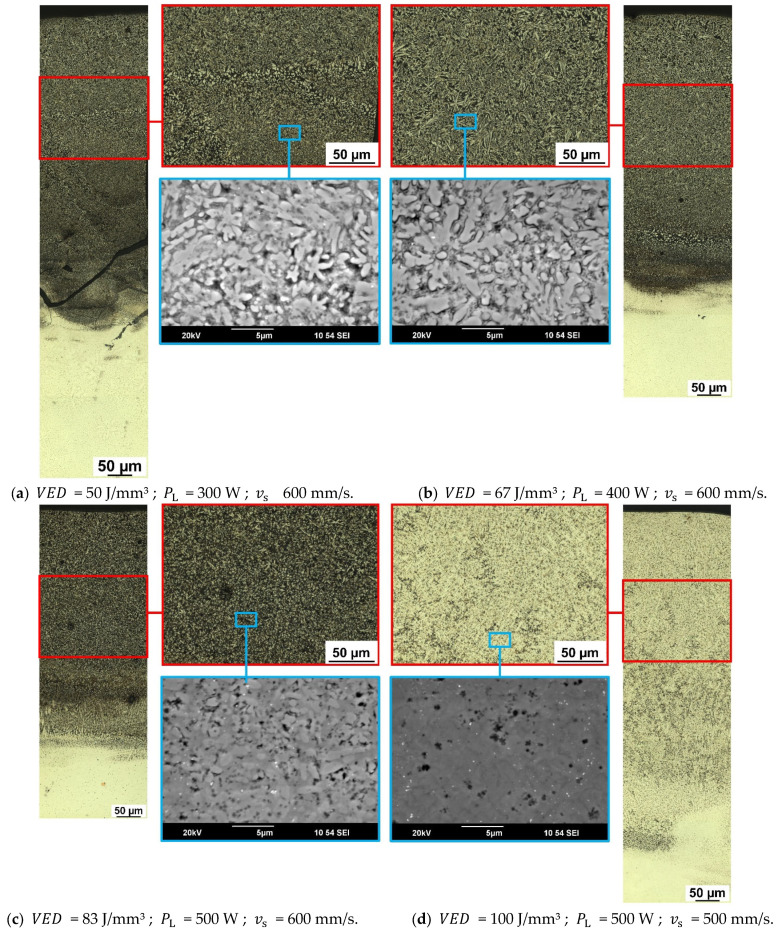
Detailed microstructural analyses, including stitched micrographs from the substrate plate to the top layer, the steady-state region (marked in red), and high-resolution SEM images (marked in blue) of: (**a**) MPEA_50, (**b**) MPEA_67, (**c**) MPEA_83, (**d**) MPEA_100.

The etched microstructures of the investigated samples are depicted in Figure 8. These stitched images represent the microstructural transition from the remelted substrate plate to the top layer of the MPEA samples fabricated via PBF-LB. An initial change in the microstructure from the substrate plate upwards is clearly visible in Figure 8. This zone—called the transition zone—in which the alloy composition changed from the substrate plate composition to the MPEA alloy, varied in thickness and corresponded to the remelting depths. Thus, the higher the VED, and with it the penetration depth into the substrate plate, the longer it took to achieve a steady-state. This steady-state is marked in red (Figure 8) and is the section investigated in detail, as it most closely represents bulk material properties. The top layer shows a significantly finer microstructure due to the absence of further remelting (overlaps) and reheating cycles. Overall, dendritic structures were predominant, and the variations in coloration post-etching enabled us to see both the mixing with the substrate plate and the differences between the samples. Most notably, the MPEA_100 sample was overall brighter in coloration and reacted to the etching in much the same way as the substrate plate itself. Since the main difference in chemical composition was the lack of Ti in the substrate plate, this strongly suggested a loss of Ti due to evaporation during PBF-LB fabrication.

**Figure 9:**
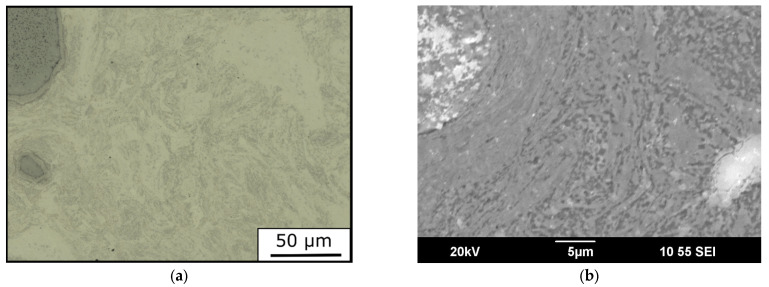
Micrographs of the MPEA_HIP sample: (**a**) light optical image, and (**b**) secondary electron image.

Micrographs of the solid-state sintered MPEA_HIP sample are depicted in Figure 9. The microstructure of the MPEA_HIP sample differed significantly from the microstructures of the four investigated PBF-LB samples (Figure 8). The compacted structure of the mechanically alloyed powder remained visible. In contrast, the feedstock was completely remelted in the PBF-LB process, thereby creating an entirely new solidification microstructure.

#### 3.2.2. Chemical Composition

**Figure 10:**
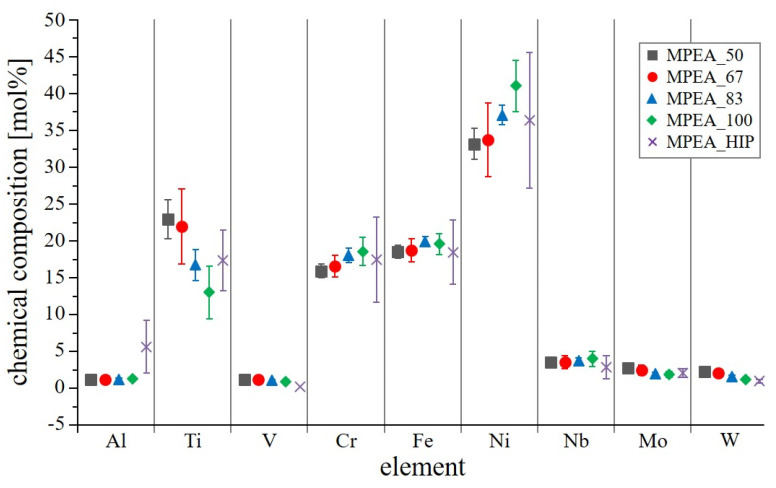
Average chemical composition of samples detected by EDS-line scans within the steady-state region.

EDS analyses within the steady-state region showed that the samples fabricated with a VED ranging from 50 J/mm^3^ to 83 J/mm^3^ were similar in their chemical composition, whereas the MPEA_100 sample suffered a distinct loss of Ti (Figure 10). This finding aligned with the reaction to the etchant, whereby the MPEA_100 sample behaved similarly to the substrate plate, which was also low in Ti. The observed drop in Ti content of MPEA_100 was due to evaporation loss and was linked to the high VED, i.e., due to the combination of a high laser power and a low scan speed setting. The MPEA_83 sample also showed a slight loss of Ti.

It should be noted that the chemical compositions of the samples did not completely match that of the CrFeNiTi MPEA (Table 1) for various reasons. The listed CrFeNiTi MPEA composition is the nominal composition based on the powder blend prior to the mechanical alloying. Thus, it does not account for losses due to sieved out flakes or the powder stuck to the milling chamber or grinding balls. Additionally, the mechanical alloying equipment is subject to wear and hence, traces of tungsten were present in the mechanically alloyed powder.

### 3.3. Micro-Hardness and Phase Fractions

#### 3.3.1. Average Hardness

**Figure 11:**
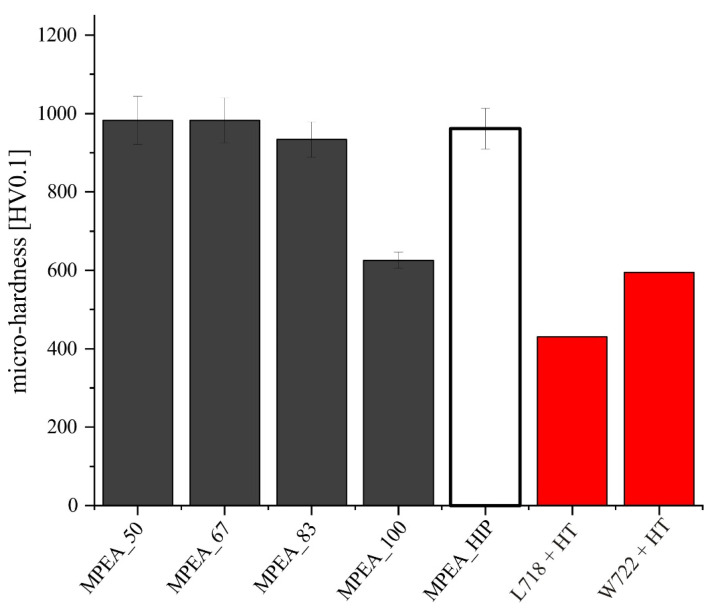
Average micro-hardness of the CrFeNiTi MPEA fabricated via PBF-LB (steady-state region) at varying VED and via solid-state sintering in an HIP process. Comparison to the well-established alloys L718 and W722 after their standard heat treatment [13,15,31].

**Figure 12:**
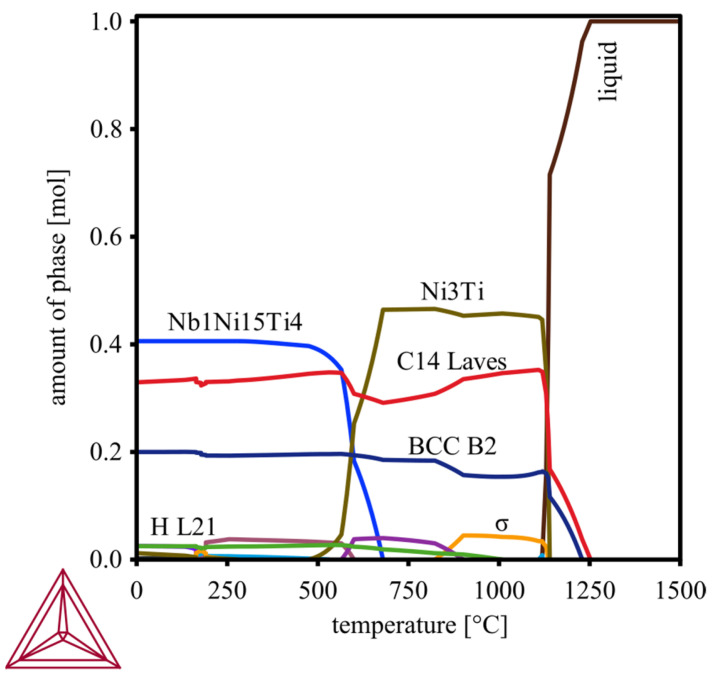
Phases and phase fractions (in mol) plotted over the temperature range 0 °C to 1500 °C for the CrFeNiTi MPEA, according to the Thermocalc calculation.

The average micro-hardness of the PBF-LB fabricated samples MPEA_50 (983 ± 62 HV0.1), MPEA_67 (983 ± 57 HV0.1), and MPEA_83 (934 ± 45 HV0.1) was comparable to the micro-hardness of the HIP sample, MPEA_HIP (961 ± 52 HV0.1). In contrast, the MPEA_100 sample showed a drastically reduced micro-hardness of only 626 ± 21 HV0.1, which is a 30% reduction in hardness (Figure 11). The main reason for this deviation was the different chemical composition of the MPEA_100 sample, which directly impacted the proportions of microstructural phases formed. The amount and formation of these phases, calculated by Thermocalc for the nominal CrFeNiTi MPEA alloy composition (Table 1), are strongly influenced by temperature (Figure 12). Calculations by Thermocalc predict a bcc_B2 phase (Cr-rich), as well as a σ-phase (FeCr). According to the Thermocalc calculation, the σ-phase is present in the temperature range of 800 °C to 1100 °C. Other phases that occur, according to the Thermocalc calculations, are an H_L21 phase (Ni_2_AlTi), the intermetallic compound Ni_3_Ti, and the ternary intermetallic compound Nb_1_Ni_15_Ti_4_ [28].

The influence of the reduced Ti content of the MPEA_100 sample on the phase fractions can be reconstructed. In the nominal state, CrFeNiTi exhibits large amounts of the C14_Laves phase in the temperature range up to ~1100 °C. The C14_Laves phase is composed of Fe and Ti (Fe_2_Ti), having a hardness ranging from 802 HV0.1 to 883 HV0.1 [29], but which can reach as high as 1242 HV [30]. Therefore, the C14_Laves phase contributed significantly to the high hardness of the CrFeNiTi MPEA. Thus, the reduced Ti content in the MPEA_100 sample coincided with a smaller amount of the C14_Laves phase, which resulted in a decrease in hardness.

In comparison to the precipitation hardened alloys L718 and W722, the hardness of CrFeNiTi is significantly higher (Figure 11) [13,15,31]. The reason for the high hardness of CrFeNiTi is the extensive solid solution strengthening, which can be exploited due to the high configurational entropy of the system [32]. In addition, the fine distribution of multiple phases strengthened the material, as was also reported in previous studies [33,34].

#### 3.3.2. Hardness Gradient

**Figure 13:**
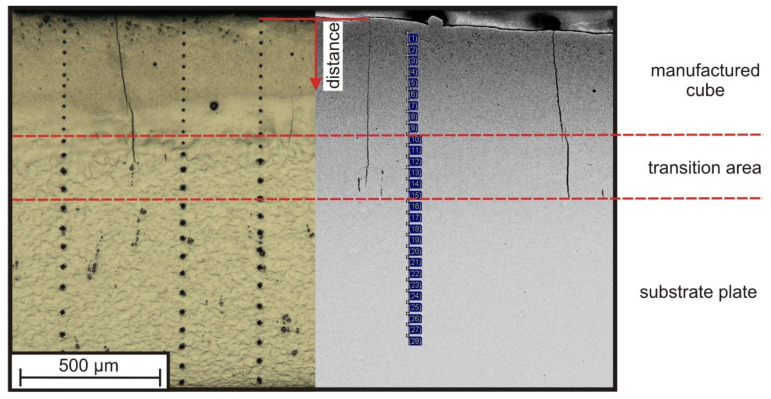
Exemplary hardness imprints (**left**) and measurement points of the EDS-line scan (**right**). The measurement series started close to the top surface of the samples and continued along a straight line parallel to the PBF-LB built direction (z-axis) to the substrate plate.

The progression of the micro-hardness was determined by a series of indentations performed on the cross-sectional cuts of the PBF-LB samples (Figure 13). In addition, EDS line scans were taken to compare the hardness gradient with the change in chemical composition, starting from the samples’ top layer through the transition zone into the substrate plate. In both cases, the reference is with respect to the distance to the top layer of each sample and parallel to the PBF-LB built direction (*z*-axis).

**Figure 14:**
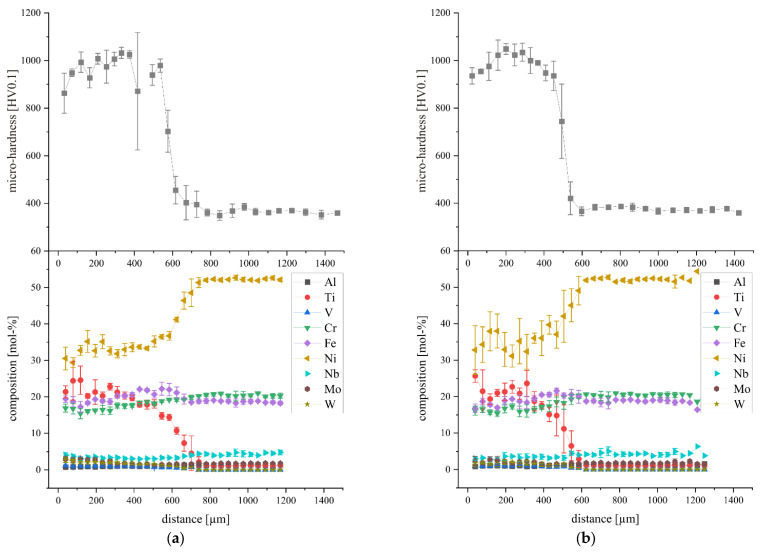

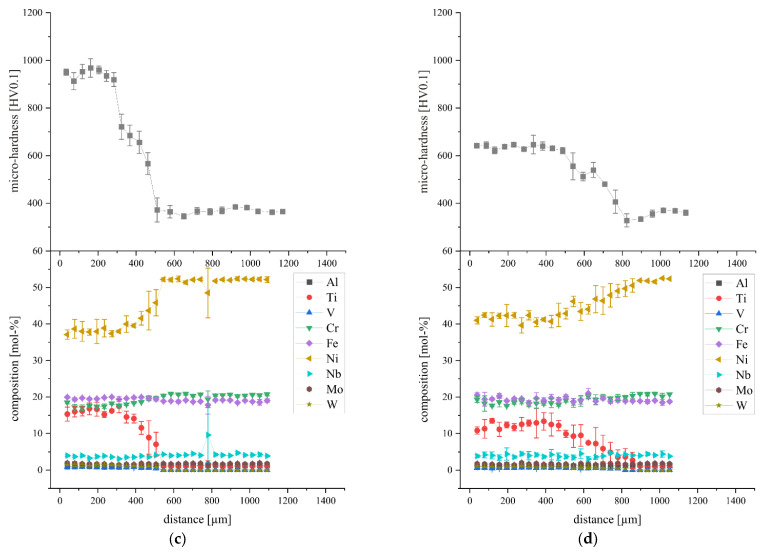
Micro-hardness and chemical composition plotted over the distance to the samples’ top surface: (**a**) MPEA_50, (**b**) MPEA_67, (**c**) MPEA_83, (**d**) MPEA_100; measuring schematic shown in Figure 13.

Two distinct areas with different hardness levels can be identified: the steady-state region within the fabricated sample (high hardness), and the substrate plate itself (Figure 14). In between these two is the transition zone, which is far more pronounced for higher VED settings. As an example, the detectable hardness transition of samples MPEA_50 and MPEA_67 was in the range of approximately 150 µm. The hardness transition of the MPEA_83 sample was about 200 µm and the MPEA_100 sample exhibited the maximum hardness transition of over 300 µm. This behavior aligned well with the remelting depths, depending on the VED settings. The deeper the melt pool penetrated into the substrate plate, the more layers were needed to reach the steady-state.

In all four cases, the progression of the micro-hardness was in good agreement with the change in the chemical composition, starting from the composition of the substrate plate to the composition of the CrFeNiTi MPEA, albeit with the exception of the MPEA_100 sample, which suffered from a significant evaporation loss regarding Ti.

## 4. Summary and Conclusions

CrFeNiTi MPEA was successfully processed via PBF-LB and solid-state sintering of a mechanically alloyed powder feedstock. The work focused on the PBF-LB fabrication route, and the solid-state sintered sample served as a control. The custom-made PBF-LB machine system is suitable for the study of HEAs/MPEAs due to the flexible design and the processing of small powder quantities. The CrFeNiTi MPEA showed a decent processability via PBF-LB within a VED of 50 J/mm^3^ to 100 J/mm^3^. Significant evaporation loss of titanium, the most volatile alloying element in the chosen mixture, only occurred at the maximum VED of 100 J/mm^3^. However, small evaporation losses already occurred at a VED of 83 J/mm^3^. The micro-hardness of the CrFeNiTi MPEA was similar for both fabrication routes and was about 965 HV0.1 on average, with a peak hardness of about 1040 HV0.1.

The study was subject to a few limitations, the foremost of which was the absence of pre-alloyed powder of the desired chemical composition. Thus, mechanical alloyed powder was used instead, which had a few drawbacks of its own. Only small powder quantities with comparatively coarse particle sizes could be established. Therefore, a small PBF-LB machinery able to cope with these conditions was employed, but it lacked the ability to preheat the fabrication chamber. Despite these difficulties, however, the CrFeNiTi MPEA was successfully processed by means of PBF-LB, and samples with large defect-free sections were produced. This serves as a good indicator that this type of alloy is suitable for the PBF-LB process, and with better processing circumstances, significantly larger, defect-free samples should be attainable.

## Figures and Tables

**Table 1:** Chemical composition of the input material powders (nominal), the mechanically alloyed CrFeNiTi MPEA (nominal), and the substrate plate (optical emission spectrometer).

Sample	Cr	Fe	Ni	Ti	Al	Co	Mo	Nb
Cr [ma.-%]	99.24	0.14	0.00	0.00	0.38	0.00	0.00	0.00
Ti [ma.-%]	0.00	0.20	0.00	99.50	0.00	0.00	0.00	0.00
W722 [ma.-%]	0.00	66.90	18.00	1.00	0.00	9.25	4.85	0.00
L718 [ma.-%]	18.00	18.50	53.68	0.95	0.50	0.00	3.00	5.30
CrFeNiTi MPEA [mol%]	20.00	20.00	34.70	20.00	0.66	1.07	1.46	2.03
Substrate plate [ma.-%]	20.65	20.86	50.2	0.9	0.48	0.3	2.78	2.76

## Data Availability

Not applicable.
